# Ascites’ neutrophil function is significantly impaired in patients with decompensated cirrhosis but can be restored by autologous plasma incubation

**DOI:** 10.1038/srep37926

**Published:** 2016-12-05

**Authors:** Cornelius Engelmann, Christina Becker, Andreas Boldt, Toni Herta, Albrecht Boehlig, Katrin Splith, Moritz Schmelzle, Niklas Mueller, Sandra Krohn, Hans-Michael Tautenhahn, Michael Bartels, Ulrich Sack, Thomas Berg

**Affiliations:** 1 University Hospital Leipzig; Section of Hepatology; Department of Internal Medicine, Neurology, Dermatology, Liebigstrasse 20, 04103 Leipzig, Germany; 2 University Hospital Leipzig; Institute of Clinical Immunology, Johannisalle 30, 04103 Leipzig, Germany; 3 Charité - Universitätsmedizin Berlin; Department for General, Visceral and Transplantation Surgery, Augustenburger Platz 1, 13353 Berlin, Germany; 4 University Hospital of Leipzig; Department for General, Visceral and Transplantation Surgery, Liebigstrasse 20, 04103 Leipzig.

## Abstract

Systemic immune cell dysfunction is a typical feature of liver diseases and increases the risk of bacterial infection, especially spontaneous bacterial peritonitis. We evaluated functional properties of neutrophil granulocytes in blood and ascites of patients both with and without decompensated cirrhosis. We collected blood and ascites samples from 63 patients with cirrhosis and eight without cirrhosis. Phagocytosis activity (PA) and oxidative burst activity (OBA) were evaluated after *ex vivo* stimulation with *E. coli*, while fluorescence signals were measured by flow cytometry. Ascites’ neutrophil function tests were repeated after incubation with autologous plasma. Ascites’ neutrophils showed an impaired PA and OBA (median blood PA 98.1% (86.8–99.8) vs. ascites’ PA 50.5% (0.4–97.3), p < 0.0001; median blood OBA 98.7% (27.5–100) vs. ascites’ OBA 27.5% (0.3–96.7), p < 0.0001). Patients with non-cirrhotic ascites showed higher PA but equally suppressed OBA. Ascites’ neutrophil function could be partially restored after incubation with autologous plasma (median increase PA: 22.5% (−49.7 – +93.2), p = 0.002; OBA: 22.8% (−10.4 – +48.8), p = 0.002). Ascites’ neutrophils of patients with cirrhosis are functionally impaired, but could be partially restored after incubation with plasma. Further investigations are needed to identify the factors in ascites that are associated with neutrophils’ function.

Patients with end-stage liver diseases typically express features of a dysfunctional immune system that are associated with a suppressed response of peripheral blood neutrophils to invading pathogens^1^,^2^,^3^. This is considered to be part of a general immune exhaustion induced by the continuous intestinal, bacterial, translocation-mediated immune stimulation in cirrhosis^4^,^5^,^6^,^7^,^8^. It is assumed that there is a strong causal relationship between so-called immune paralysis and the high rate of infectious complications in decompensated liver cirrhosis^9^,^10^,^11^,^12^. To date, however, it is unclear why ascites or peritoneal cavities are the predominant site of bacterial infection in patients with decompensated cirrhosis (that is, spontaneous bacterial peritonitis (SBP)), while this type of infection is only rarely seen in patients with malignant ascites^13^,^14^.

Studies that specifically address peritoneal host defence mechanisms in decompensated cirrhosis cases are few and far between. The phagocytosis and oxidative burst capacity of peritoneal macrophages has been found to be severely impaired^15^, and the level of opsonic activity in ascites has been linked to the risk of developing SBP^14^,^15^. Only one study has evaluated functional properties in ascites’ neutrophils^16^,^17^, by comparing phagocytosis and oxidative burst activity in patients with and without SBP. However, the function of the peripheral blood neutrophil counterpart was not studied, so it remains a matter of speculation whether the findings in ascites are simply a reflection of the systemic neutrophil dysfunction that has been observed in patients with liver failure.

Due to this lack, we were interested in whether neutrophils in ascites of patients with decompensated cirrhosis show a higher degree of functional impairment, not only compared to their blood counterparts, but also to ascites’ neutrophils derived from patients with non-cirrhotic ascites.

## Results

### Phagocytic and oxidative burst rate of neutrophils derived from patients with cirrhosis

Neutrophil function was determined by flow cytometry after stimulation with inactivated and opsonised *E. coli* bacteria. Phagocytic rate and oxidative burst rate were determined as the percentage of active neutrophils in relation to the total number of viable neutrophils. Phagocytosis could be determined in 62 out of 63 blood samples and in 60 out of 63 ascites samples from patients with cirrhosis. Oxidative burst was measurable in all (63/63) blood samples and in 62 out of 63 ascites samples.

**Figure 1:**
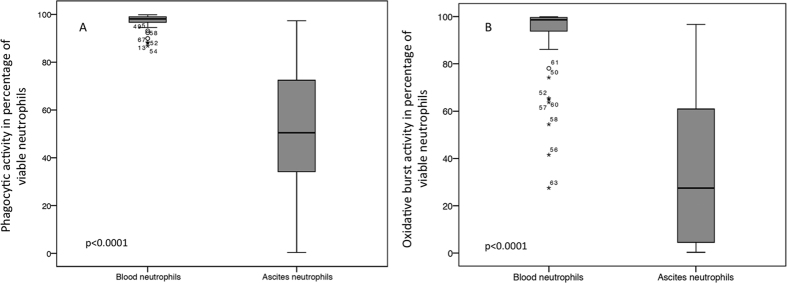
Phagocytic rate (**A**) and oxidative burst rate (**B**) of neutrophils in blood and ascites. Boxplots show that neutrophils’ function was significantly diminished in ascites’ neutrophils, compared to blood neutrophils. Values are given as the percentage of viable neutrophils.

The median ascites’ phagocytic rate was 50.5% (range 0.4–97.3), compared to 98.1% (range 86.8–99.8; p < 0.0001) in blood neutrophils. The median ascites’ oxidative burst rate was 27.5% (range 0.3–96.7), compared to 98.7% (range 27.5–100; p  < 0.0001) in blood (see Fig. 1). The ascites’ neutrophil functions were not correlated with the functioning of blood neutrophils (correlation coefficient for phagocytic rate: r = 0.213 (p = 0.102), and for oxidative burst rate: r = 0.165 (p = 0.2)). In addition, the ranges of phagocytic and oxidative burst rates were broader in ascitic fluid than in blood neutrophils, ranging from normal to nearly undetectable rates (see Fig. 1), which possibly indicates that additional environmental factors may be involved in the mechanisms of peritoneal neutrophil stimulation.

### Neutrophil function in patients with non-cirrhotic ascites

**Figure 2:**
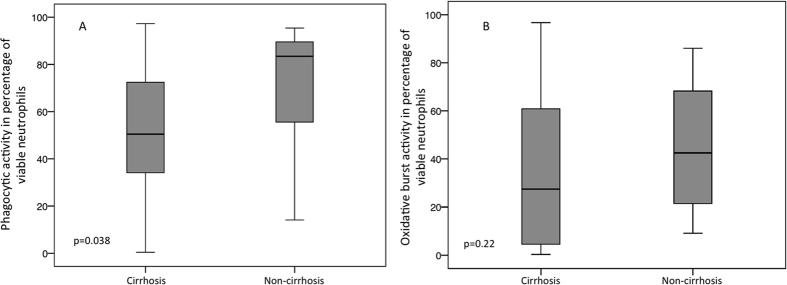
Phagocytic rate (**A**) and oxidative burst rate (**B**) of ascites’ neutrophils, presented separately for cirrhosis and non-cirrhosis. Boxplots show that phagocytosis activity, but not oxidative burst activity, in ascites’ neutrophils was reduced in cirrhosis compared to non-cirrhotic ascites. Values are given as the percentage of viable neutrophils.

The median phagocytic rate of neutrophils in non-cirrhotic ascites was 83.5% (range 14.1–95.4), 33% higher than in ascites’ neutrophils of patients with cirrhosis (p = 0.038) (see Fig. 2). The median ascites’ neutrophil oxidative burst rate was 42.5% (range 9.1–86). Although the neutrophils increased by about 15% in cirrhotic ascites, they did not reach statistical significance (p = 0.22). The ascites’ protein level was the major factor differentiating ascitic fluid in cirrhotic and non-cirrhotic patients, being significantly higher in the latter group (median ascites’ protein content in cirrhosis was 13.2 g/L (range 0–58.4) vs. 23.55 g/L (range 21.4–46) in non-cirrhosis, p = 0.001).

The blood neutrophils of patients without cirrhosis showed a median phagocytosis rate of 99% (97.2–99.8), 1.8% lower than in the cirrhosis group (p = 0.049). The median oxidative burst rate, with a median of 98.5% (62.7–100) was not different in blood neutrophils in cirrhosis (−0.2%, p = 0.792) compared to patients with cirrhosis.

### Factors associated with ascites’ neutrophil function in cirrhosis

We further assessed whether patient characteristics, as well as markers of liver disease severity, correlated with neutrophil function in ascites. Although male patients exhibited higher phagocytosis and oxidative burst activity than their female counterparts, the difference did not reach statistical significance (median phagocytic rates in males was 60.5% (range 3.3–96.3) vs. 35.7% (range 0.4–97.3) in females, p = 0.065; median oxidative burst rates in males was 28.8% (range 0.3–96.7) vs. 9.7% (range 0.5–89.2) in females, p = 0.18). In addition, for all other parameters, including age, body weight, Child-Pugh score, model of end-stage liver disease (MELD) score, white blood cell count (WBC), C-reactive protein and medical treatment, no clear correlation with the level of ascites’ neutrophil function could be found (see Supplementary Table 1, Supplementary Figure 3 and Supplementary Figure 4).

**Figure 3:**
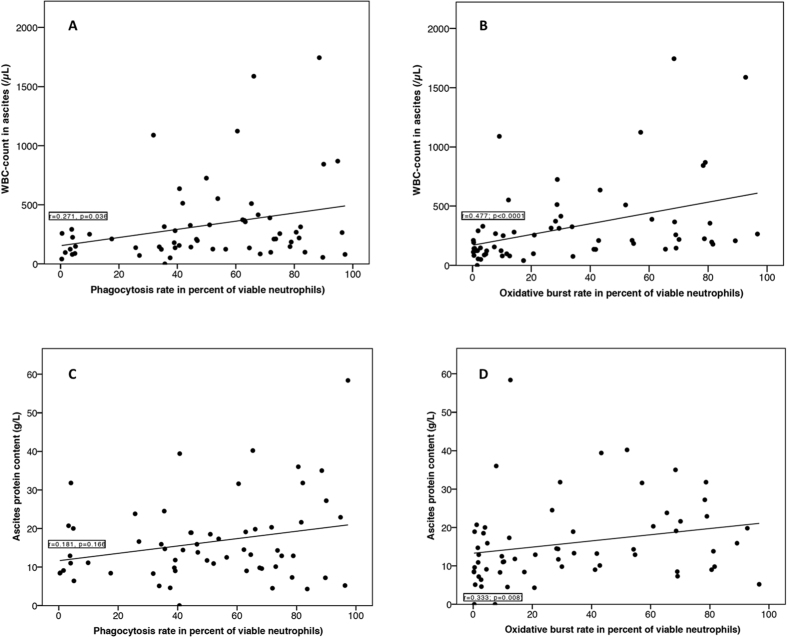
Ascites’ neutrophils’ functions in patients with cirrhosis in correlation with the ascites’ leukocyte count and protein content. Figure (**A**) shows the correlation between the phagocytic rate and WBC count in ascites; figure (**B**) between the oxidative burst rate and the WBC count in ascites’ figure (**C**) between the phagocytic rate and ascites’ protein content; and figure (**D**) between the oxidative burst rate and ascites’ protein content. For correlation analysis, the Spearman-Rho coefficient was calculated and a correlation coefficient of r > 0.5 was considered relevant.

The ascites’ leukocyte count showed a weak association with the oxidative burst rate (leukocyte count: r = 0.477, p < 0.0001) but not with the phagocytic rate (r = 0.271; p = 0.036) in ascites’ neutrophils (see Fig. 3). In patients with cirrhosis and SBP, the median oxidative burst rate was significantly higher than in those without SBP (median 48.1% (9.1–92.7) vs. median 19% (0.3–96.7, p = 0.014). Concerning the phagocytosis rate, no significant difference in relation to SBP was observed (SBP: median 62.9% (31.6–94.8) vs. no SBP: median 48.9% (0.4–97.3), p = 0.150).

Added to this, the ascites’ protein content showed neither an association with the phagocytic rate (r = 0.181, p = 0.166) nor with the oxidative burst rate (r = 0.333, p = 0.008) (see Fig. 3).

### Factors associated with circulating neutrophil function in cirrhosis

There was no clear correlation between functional properties of blood neutrophils and baseline parameters such as age, body weight, Child-Pugh score, serum protein and albumin (Supplementary Table 2). Although oxidative burst rate showed a negative correlation with the MELD score (r = −0.278, p = 0.028), WBC (r = −0.27, p = 0.032) and C-reactive protein (r = −0.34, p = 0.022) the coefficient did not meet our criteria for a valid correlation (i.e. r > 0.5). However, in patients with more advanced decompensation of liver function defined by a MELD score ≥ 15 or a cirrhosis stage Child-Pugh C the oxidative burst rate was significantly reduced as compared to patients with more preserved liver function (MELD ≥ 15: median oxidative burst rate 19% (range 0.3–89.2) vs. MELD < 15: 43.8% (range 0.3–96.7). p = 0.023) or Child-Pugh C category (Child C: median oxidative burst rate 9.7% (range 0.3–80.6) vs. Child B: 41% (range 0.3–96.7) vs. Child A: 38.5% (range 2.8–79) p = 0.026).

### Ascites’ neutrophil function was partially restored after incubation with autologous plasma

We artificially modulated environmental conditions for ascites’ neutrophils *ex vivo* via incubation with autologous plasma.

**Figure 4:**
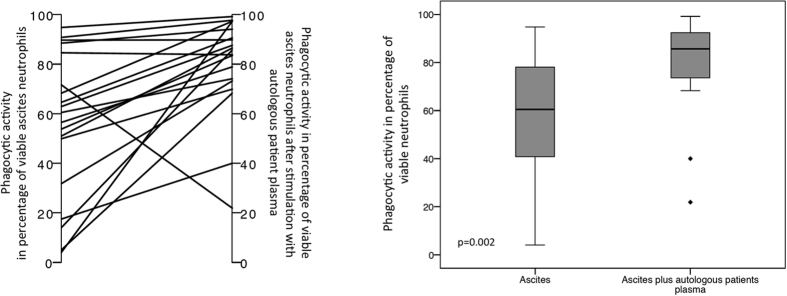
Phagocytic rate of ascites’ neutrophils after incubation with autologous plasma. The left plot depicts the individual changes, while the boxplots show the distribution of phagocytic rates of ascites’ neutrophils before and after incubation with autologous patients’ plasma. Values are given as the percentage of viable neutrophils.

**Figure 5:**
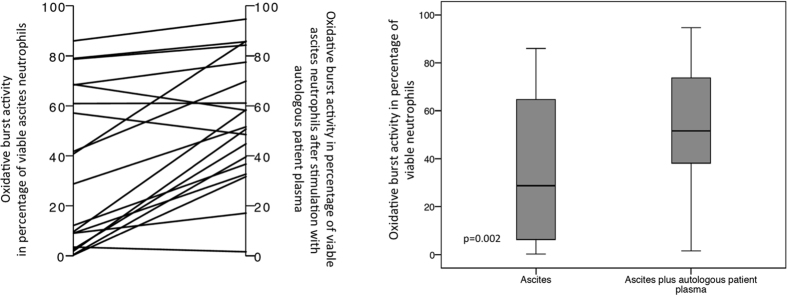
Oxidative burst rate of ascites’ neutrophils after incubation with autologous plasma. The left plot portrays the individual changes, while the boxplots present the distribution of oxidative burst rates of ascites’ neutrophils before and after incubation with autologous patients’ plasma. Values are given as the percentage of viable neutrophils.

Ascites neutrophils’ showed a significant net increase in median phagocytic activity, by 22.5% (range −49.7–93.2) (from 60.5% (range 4.1–94.8) to 85.7% (range 21.9–99.2), p = 0.002) (see Fig. 4) after said procedure. However, it did not result in a full restoration of phagocytic activity, which was still lower in ascites’ neutrophils than in blood neutrophils’ median phagocytic rate in the blood (97.4% (range 86.8–99.8, p < 0.0001)). Similarly, although oxidative burst activity did increase after *ex vivo* plasma incubation, with a median net increase of 22.8% (range −10.4–48.8) (median oxidative burst rate 28.8% (range 0.3–86) vs. 51.6% (range 1.6–94.7), p = 0.002) (see Fig. 5), it also did not reach the activity levels obtained in blood neutrophils (median oxidative burst rate 96% (range 27.5–100), p = 0.001).

A total of five patients did not show any response to plasma incubation, but actually presented a worsening of phagocytosis (n = 2) and oxidative burst (n = 3); only one of these suffered from non-cirrhotic ascites.

## Discussion

Immune paralysis in general, and neutrophil dysfunction in particular, are common phenomena in chronic liver diseases and increase the risk of infectious complications^18^. SBP is by far the most common bacterial infection in this context. This is in contrast to malignant ascites, where SBP is a rare event^14^, possibly indicating differences in the response to invading pathogens. Although it has been suggested that the peritoneal cavity in cirrhosis might be a privileged site with specifically reduced host defence mechanisms^19^,^20^,^21^,^22^, there have been limited studies evaluating ascites’ neutrophil function as a potential contributory factor to the specific susceptibility **o**f the peritoneal cavity to bacterial infections. We therefore evaluated phagocytosis and oxidative burst activity in both ascites and blood neutrophils derived from patients with and without cirrhosis.

In a significant cohort of 63 patients with decompensated cirrhosis, we showed for the first time that, when compared with peripheral blood neutrophils, ascites’ neutrophils were severely dysfunctional, showing a reduction of phagocytosis and oxidative burst rates of approximately 50% and 70% respectively. So far, only one study^16^ has investigated the functional properties of ascites’ neutrophils, looking at 9 and 19 patients with and without SBP. The presence of SBP was associated with lower neutrophil oxidative burst activity, as compared to neutrophils derived from non-infected ascites samples, but activity increased during antibiotic treatment. However, neither the phagocytic rate nor the functional properties of the peripheral blood neutrophils have been studied.

In comparison to previous reports, the functional properties of blood neutrophils in cirrhosis were found to be less depressed in our cohort, which might be, however, partly explained by a relative underrepresentation of patients with heavily impaired liver function^18^,^23^. Indeed, we could confirm a significantly reduced blood neutrophils oxidative burst rate when only patients with more advanced decompensation of the liver function (i.e. Child C and MELD ≥ 15) were analysed.

An intriguing finding of our study was the high degree of variability of ascites’ neutrophil function, ranging from 0.4% to 97.3% for phagocytosis and from 0.3% to 96.7% for oxidative burst. This result was in contrast to the results obtained from the blood-derived neutrophils, in which the activity rates were more homogeneous, ranging from 86.8% to 99.8% (phagocytosis) and from 27.5% to 100% (oxidative burst). This variability of ascites’ neutrophil activity could not be explained by certain patient characteristics, such as liver function, inflammatory parameters or age, although it is known that activation, migration and response to bacteria of circulating neutrophils depends on the degree of liver insufficiency, systemic inflammation and age^18^,^24^,^25^.

We therefore hypothesised that peritoneal cavity- and/or ascite-specific factors must exist, and are responsible for the observed site-specific differences in neutrophil function. Appropriate neutrophil activation is closely regulated by a number of stimulatory, but also inhibitory, factors such as immunoglobulins, complement factors, especially C3b, cytokines (IFN-γ, IL-8, GM-CSF and TNFα) and endotoxin levels^26^,^27^. At the time of data acquisition and the *ex vivo* experiments, we were not able to directly measure these factors. However, certain results may indicate the presence or absence of such stimulating or inhibiting factors.

We were able to stratify our results according to the ascites’ protein content and leucocyte count as well as the cause of ascites (cirrhotic vs. non-cirrhotic), all of which are well-known predictors of SBP risk^14^,^16^,^22^,^28^. We found no association between ascites’ neutrophil function and their protein content. However, because both tests – phagocytosis and oxidative burst – were performed using pre-opsonised *E. coli* bacteria, factors associated with the opsonic capacity of ascites fluid could not be studied in our test system. In contrast to the phagocytic rate, the leukocyte count in ascites had a weak, yet still significant, impact on the oxidative burst rate, being lower when the leucocyte count was high, thereby confirming the results of Nieto *et al*.^16^. The oxidative burst capacity of neutrophils derived from non-cirrhotic ascites was identical to that seen in blood neutrophils, but the phagocytosis rate was reduced by approximately 30%. As blood neutrophil function was maintained in our cohort, and neutrophils adapt to their environment, we hypothesised that the functional properties of ascites’ neutrophils may improve upon transferral to patient plasma. Indeed, phagocytosis and oxidative burst rates did recover when ascites’ neutrophils were incubated with autologous plasma, showing an increase of about 25% in both function tests. This observation is in line with results previously published by Nieto *et al*.^16^, which showed that host defence mechanisms are not irreversibly altered in decompensated liver disease, and that treatment as well as active modification of ambient conditions *in vitro* restores neutrophil function. Lebrun *et al*.^29^ were the first to show that the functional properties of immune cells can be manipulated by altering their environment. Neutrophils of healthy blood donors were brought into contact with the ascites of patients with cirrhosis (n = 32) and those of patients with malignant diseases (n = 17). Neutrophil function was assessed by chemiluminescence with pre-opsonised zymosan, a strong stimulating signal, in all samples and in four samples from a phagocytosis test using pre-opsonised *Staphylococcus aureus* cells. All functional neutrophil properties were significantly more favourable in samples derived from malignant ascites, as compared to those obtained from ascites of patients with cirrhosis. In addition, it was effectively demonstrated that a disequilibrium between inhibitory and stimulatory signals most likely contributes to the high variability of ascites’ neutrophil function. If the ascites of patients with cirrhosis were diluted with saline, their neutrophil function was partially restored. In contrast, when cirrhotic ascites were diluted with their counterparts derived from malignant ascites, their function was significantly improved. It has to be pointed out, however, that there was a lack of direct evidence for environmental factors affecting neutrophil functions. Our results may, however, stimulate further research that would elucidate the functional mechanisms that are potentially involved in neutrophil activation and migration within the micro-milieu of cirrhotic ascites.

In conclusion, this study was able to show, for the first time, that neutrophil function in ascites is severely impaired. This may explain the high susceptibility to spontaneous bacterial peritonitis in cirrhotic patients. The dysfunction of ascites’ neutrophils may be partially restored after incubation with autologous plasma. The high functional variability observed in neutrophils derived from ascites suggests that ascites’ neutrophil function is dependent on both stimulatory and inhibitory factors. Further studies are needed to clarify the individual factors involved in ascites’ neutrophil activity, as these may become potentially interesting targets for SBP treatment and prevention.

## Patients and Methods

### Study design

Between August 2014 and May 2015, ascites, fluid and corresponding blood samples were consecutively collected from 63 patients with decompensated cirrhosis at the Section of Hepatology, University Hospital Leipzig, for the purpose of evaluating the ascites’ neutrophil function by performing *in vitro* tests for phagocytosis and oxidative burst. Patients with ascites but without cirrhosis (n = 8) served as controls. All patients with ascites, who had been admitted to our hospital, were considered for participation in the study. In line with international clinical practice guidelines^29^, paracentesis was indicated for new-onset or worsening ascites and in cases where SBP was suspected. Patients with ongoing alcohol abuse, who were receiving immunosuppressive therapy or who were recovering from a liver transplant were excluded from the study. The first paracentesis after enrolment was defined as the baseline for collection of ascites and blood samples. Any subsequent paracenteses were not included in the present analysis. All clinical and laboratory data as obtained exclusively during routine visits not related to the study, was collected retrospectively. The study protocol conformed to the ethical guidelines of the 1975 Declaration of Helsinki, and was approved by the ethics committee of the University of Leipzig (No. 182 – 14 – 02062014). All patients gave written informed consent.

### Patient characteristics and data collection

The patients’ characteristics are summarised in Table 1. Alcohol abuse was the main cause of cirrhosis (71.4%) and malignancies the main reason for ascites (75%) in the non-cirrhotic group. The median MELD score was 16 points (range 6–38), and median Child-Pugh score 9 points (range 5–14), in patients with cirrhosis. After subsequent Child-Pugh classification, the majority of patients with cirrhosis were assigned to Child-Pugh class B (Class A: n = 11 (17.5%); Class B: n = 30 (47.6%); Class C: n = 22 (34.9%)). SBP was diagnosed on the basis of an elevated leukocyte count in ascites (>500/mm^3^) in 11 out of 63 patients (17.5%) with cirrhosis. At baseline, the following clinical and laboratory data was collected: cause of ascites, etiology of liver cirrhosis, drug history, sex, age, liver and renal function test, platelet count, white blood cell count, serum sodium, haemoglobin, C-reactive protein, serum albumin and protein content. Ascites samples were characterised by their total leukocyte count (via automated cell counter) and their protein content was defined according to institutional standards. SBP was diagnosed in patients with cirrhosis if the ascites’ leukocyte count was elevated above 500/mm^3^ 30.

### Ascites and blood sampling

Ascites samples were collected after careful skin disinfection and under ultrasound guidance. For local anaesthesia, 5–10 mL of Xylocaine (1%) was injected, and afterwards, a 6 French paracentesis cannula (Peter Pflugbeil GmbH, Zorneding, Germany) was inserted into the peritoneum. **An** initial fraction of 50 mL **of** ascitic fluid was used for routine laboratory analysis, while 40 mL of ascitic fluid was obtained for tests related to the study. Venous blood samples were collected under standard aseptic conditions, using a 0.8 mm Multifly-Safety needle (Sarstedt AG & Co KG, Nümbrecht, Germany) immediately after paracentesis, and decanted into heparinised tubes (18 mL) and EDTA tubes (5.7 mL). All ascites and blood samples were processed within four hours after sample collection, under pyrogen-free conditions. For antibody staining, 2 mL of ascitic fluid was poured into Eppendorf tubes (1 mL each). In preparation for flow cytometry and neutrophil function tests, the remaining 38 mL of ascitic fluid was centrifuged in a 50 mL tube at 500 × g for five minutes at room temperature. The neutrophil-containing pellet was re-suspended with 1.5 mL of ascites’ supernatant and used for the neutrophil function tests. The remaining ascites; supernatant was stored at −80 °C. The heparinised blood was processed for neutrophil function tests without prior preparation. The following steps were the same for blood and ascitic fluid (hereafter referred to as test substances).

### Flow cytometry

**Figure 6:**
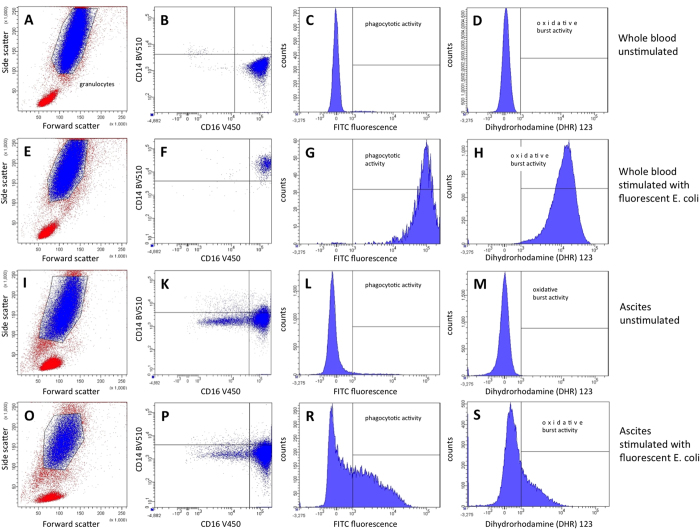
Flow cytometric analyses of phagocytosis and oxidative burst in neutrophils of patients with cirrhosis. Analysis of the neutrophils’ granulocyte functions by phagocytosis and oxidative burst in peripheral blood (**A**–**H**) and ascites (**I**–**S**). Neutrophils’ granulocytes were separated from peripheral blood (**A,E**) and ascites (**I,O**) by FSC vs. SSC and additionally characterised by surface staining with CD14 BV510 and CD16 V450 with (**F,P**) and without (**B,K**) stimulation with fluorescent *E. coli*. In peripheral blood, the expression of CD14 increased after stimulation (**F**) in contrast to neutrophils’ granulocytes in ascites (**P**), when both were compared to controls without stimulation (**B,K**). The analysis of phagocytosis and oxidative burst revealed a normal activity in peripheral blood’s neutrophils’ granulocytes (**G,H**), in contrast to a strongly attenuated activity in ascites’ neutrophils’ granulocytes (**R,S**), when both were compared to controls without stimulation (**C,D,L,M**). (**A–D**) Whole unstimulated blood. (**E–H**) Whole blood stimulated with fluorescent *E. coli*. (**I–M**) Ascites unstimulated. (**O–S**) Ascites stimulated with fluorescent *E. coli*.

Each test substance was analysed by flow cytometry using FACS CantoII DiVa software (BD Bioscience, New Jersey, USA), in order to identify neutrophils and perform neutrophil function tests (see Fig. 6). As flow cytometry in ascites is not established, the presence of neutrophils was confirmed by labelling with antibodies against CD14 (BV510; Clone: MfP9; BD Horizon), CD16 (V450; Clone: 3G8; BD Horizon), CD45 (PerCP; Clone: 2D1; BD Bioscience), CD282 (APC; Clone: TL2.1; eBioscience), CD284 (PE-Cy7; Clone: HTA125; eBioscience) and CD62L (APC-Cy7; Clone: DREG-56; BioLegend), antigens that are typically located on neutrophil granulocytes, in the first 26 patients (see Supplementary Figures 1 and 2). In total, 2.5 μL of CD14, 2.5 μL of CD16, 5 μL of CD45, 5 μL of CD282, 5 μL of CD284 and 10 μL of CD62L were added to 100 μL of the test substance and incubated for 15 minutes, while being protected from light, at room temperature. Intact immune cell subtypes were gated and identified by their characteristic forward and sideward scatter. For neutrophil function tests, cell viability could be determined by using propidium iodide, which binds to the DNA of non-viable cells. The latter, as well as artefacts, could be excluded by setting a live gate at the propidium iodide histogram. Thereafter, flow cytometry for phagocytosis and oxidative burst tests was performed as described in the following section. For the function tests, neutrophils were labelled with CD14 and CD16 antibodies (see Fig. 6).

### *In vitro* neutrophil function tests

#### Phagocytosis

The phagocytosis test was performed using the Phagotest test kit (Orpegen Pharma, Heidelberg, Germany), which contains fluorescein isothiocyanate (FITC)-labelled, opsonised and inactivated *E. coli* bacteria; samples were subsequently analysed by flow cytometry (see Fig. 6). In brief, each test substance was poured into two tubes, one for the phagocytosis test (100 μL) and one for the controls (100 μL), and cooled on ice for 10 minutes. Thereafter, 20 μL of Phagotest reagents (*E. coli* bacteria) were added to the test tubes and incubated in a shaking water bath at 37 °C for 10 minutes. The control tube remained on ice. Phagocytosis was stopped after 10 minutes with 100 μL of quenching suspension. All samples were washed twice in 3 mL of washing solution, before being centrifuged at 500 × g for 5 minutes. The supernatant was discarded. The remaining pellet was then re-suspended in 2 mL of erythrocyte lysis buffer and subsequently incubated in darkness at room temperature for 20 minutes. After centrifugation at 500 × g for 5 minutes, the supernatant containing the lysed erythrocytes was again discarded. In order to identify viable neutrophils, the pellet was re-suspended in 200 μL of DNA staining solution (propidium iodide (PI)). Flow cytometry analysis was performed within one hour. During this period, samples remained on ice in darkness.

#### Oxidative burst

Phagoburst (Orpegen Pharma, Heidelberg, Germany) contains unlabelled, opsonised *E. coli* bacteria and was used to quantify neutrophil oxidative burst (see Fig. 6). For sample preparation, 100 μL of each test substance was decanted into three tubes for the oxidative burst test, as well as for a negative and positive control. Tubes were cooled on ice for 10 minutes. 20 μL each of unlabelled, opsonised *E. coli* for the oxidative burst test, a washing buffer for the negative control, and PMA solution (phorbol 12-myristate 13-acetate) for the positive control were added to the respective tubes containing 100 μL of the test substance, and incubated at 37 °C in a shaking water bath for 10 minutes. Thereafter, 20 μL of the burst substrate (DHR123) was added to each tube, before they were incubated again at 37 °C in a shaking water bath for 10 minutes. The next steps, consisting of erythrocyte lysis and DNA staining, corresponded to the phagocytosis test and were described previously.

### *In vitro* neutrophil function test after incubation with autologous plasma

We hypothesised that environmental factors play a major role with respect to neutrophil function in ascitic fluid. Accordingly, we modified the environmental conditions *in vitro* and subsequently repeated the neutrophil function tests in the last 19 patients (cirrhotic ascites n = 16, non-cirrhotic ascites n = 3). For this purpose, patients’ ascites’ neutrophils were incubated with autologous plasma. Heparinised blood was centrifuged at 2000 × g at room temperature for 5 minutes. 300 μL of the resulting plasma (supernatant) was mixed with 300 μL of the re-suspended ascites pellet. Following this, the phagocytosis and oxidative burst tests were performed as previously described.

## Statistical analysis

Statistical analysis was performed using SPSS 22 software (SPSS Inc., Chicago, IL). Categorical variables were displayed as percentages or frequencies, and continuous variables as mean ± standard deviation or median and range, as appropriate. A two-sided p-value of < 0.05 was considered statistically significant. Comparison of unpaired samples was performed by Mann-Whitney U test in the case of continuous data and by Chi-square test for discrete data. Paired samples were compared by Wilcoxon signed rank test. For correlation analysis, the Spearman-Rho coefficient was calculated and a correlation coefficient of r > 0.5 was considered relevant.

## Additional Information

**How to cite this article**: Engelmann, C. *et al*. Ascites’ neutrophil function is significantly impaired in patients with decompensated cirrhosis but can be restored by autologous plasma incubation. *Sci. Rep.* **6**, 37926; doi: 10.1038/srep37926 (2016).

**Publisher's note:** Springer Nature remains neutral with regard to jurisdictional claims in published maps and institutional affiliations.

## Supplementary Material

Supplementary Information

## Figures and Tables

**Table 1:** Patient characteristics at baseline paracentesis.

Variable	Cirrhosis (n = 63)	Non-cirrhosis (n = 8)	Level of significance (p)
Age (years), mean ± SD	59.5 ± 10.3	62.9 ± 17.4	0.233
Gender (male/female), n (%)	45/18 (71.4%/28.6%)	5/3 (62.5%/37.5%)	0.053
Etiology of cirrhosis, n (%)		Not applicable	
Alcoholic	45 (71.4%)		
NASH	5 (7.9%)		
Cryptogenic	9 (14.3%)		
Others	4 (6.3%)		
Cause of ascites (non- cirrhosis), n (%)	Not applicable		
Malignant		6 (75%)	
Cardiogenic		1 (12.5%)	
Acute BCS		1 (12.5%)	
Bilirubin (μmol/L), median (range)	29.6 (4–541)	10.4 (3.6–20.9)	0.0001
Albumin (g/L), median (range)	31.9 (13.9–49.5)	33.1 (25.9–54.5)	0.499
INR, median (range)	1.5 (0.9–3.5)	1.1 (0.9–1.53)	0.012
Serum creatinine (μmol/L), median (range)	106 (41–389)	78 (31–107)	0.018
GFR (ml/min), median (range)	60 (10–119)	67 (51–107)	0.483
Thrombocyte count (exp9/L), median (range)	86.5 (29–332)	320 (127–474)	0.001
White blood cell count (exp9/L), median (range)	5.8 (2.9–30.3)	7.05 (2.4–12.6)	0.617
Haemoglobin (mmol/L), median (range)	6.6 (4–9)	6.1 (5.1–7.6)	0.859
C-reactive protein (mg/dL), median (range)	18.9 (1.01–140.31)	16.2 (9.2–125)	0.986
Total protein content blood (g/L), median (range)	63.1 (44.1–78.8)	64 (53.5–64.3)	0.635
Total protein content ascites (g/L), median (range)	13.2 (0–58.4)	23.6 (21.4–46)	0.001
Ascites leukocyte count (/mm^3^), median (range)	210 (0–1744)	346.5 (148–2715)	0.036

Categorical data is displayed as absolute and relative values and metric data as mean ± standard deviation or median (range), as appropriate.

NASH = non-alcoholic steatohepatitis.

BCS = Budd-Chiari syndrome.

GFR = glomerular filtration rate.

INR = international normalised ratio.

SD = standard deviation.
