# Supplementary material for: Ascites’ neutrophil function is significantly impaired in patients with decompensated cirrhosis but can be restored by autologous plasma incubation
Source: Sci Rep. 2016 Dec 5;6:37926. doi: 10.1038/srep37926 (PMC5137075; doi:10.1038/srep37926)
Supplement: Supplementary Information [file srep37926-s1.pdf]

Ascites neutrophil function is significantly impaired in patients with decompensated cirrhosis but can be restored by autologous plasma incubation

Cornelius Engelmann[1+*], Christina Becker[1,2+], Andreas Boldt[2], Toni Herta[1], Albrecht Boehlig[1], Katrin Splith[3], Moritz Schmelzle[3], Niklas Mueller[1], Sandra Krohn[1], Hans-Michael Tautenhahn[4], Michael Bartels[4], Ulrich Sack[2#], Thomas Berg[1#]

[+]Contributed equally

[#] Contributed equally

Supplementary Material

Figures

Supplementary Figure 1:

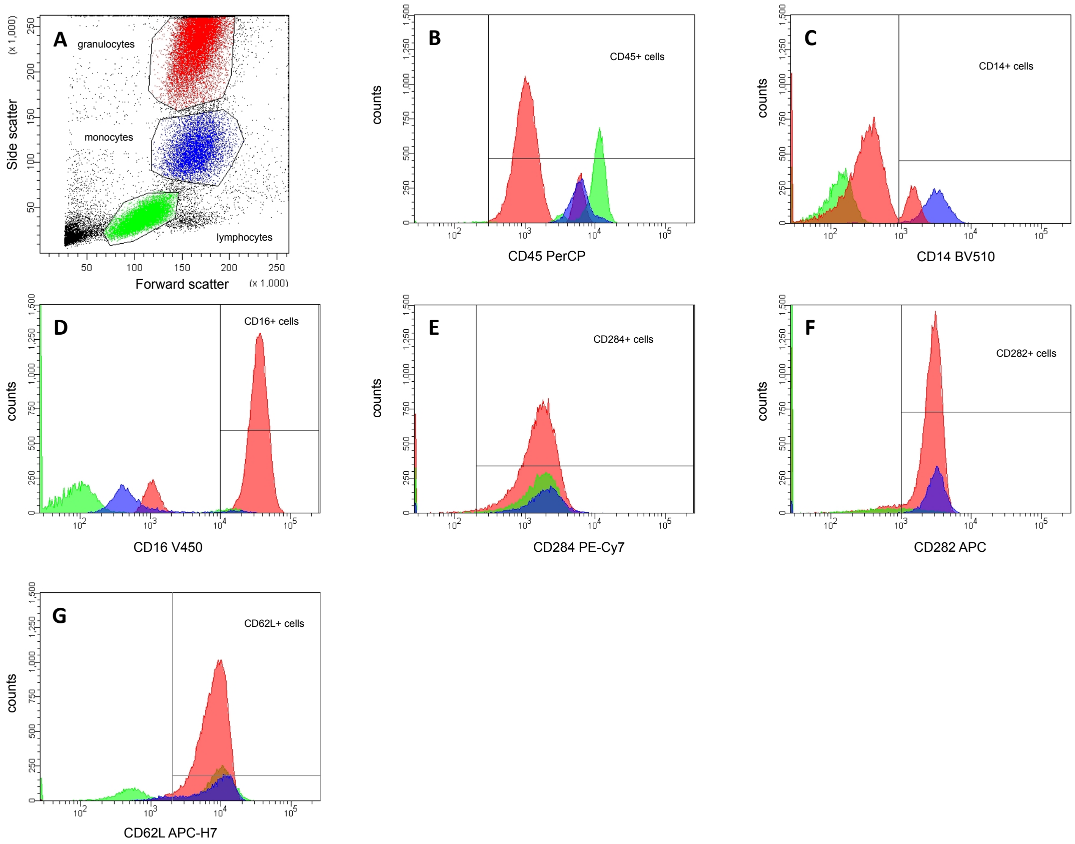

Exemplary flow cytometric characterization of granulocytes (A, red color) in peripheral blood by surface markers typically located on granulocytes: CD45 (B), CD14 (C, only on activated granulocytes), CD16 (D), CD284 (E), CD282 (F) and CD62L (G). Especially staining with CD14 (C) and CD16 (D) can be used to differentiate granulocytes from lymphocytes (A, green color) and monocytes (A, blue color).

Supplementary Figure 2:

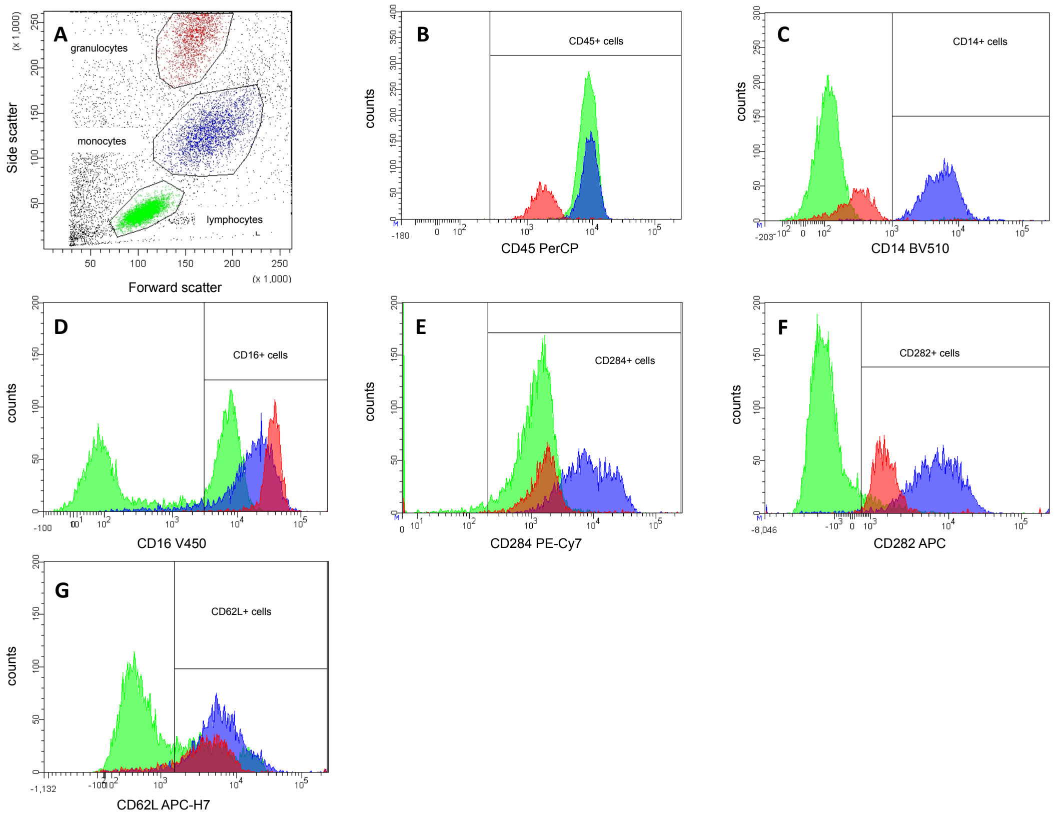

Exemplary flow cytometric characterization of granulocytes (A, red color) in ascites by surface markers typically located on granulocytes: CD45 (B), CD14 (C, only on activated granulocytes), CD16 (D), CD284 (E), CD282 (F) and CD62L (G). Especially staining with CD14 (C) and CD16 (D) can be used to differentiate granulocytes from lymphocytes (A, green color) and monocytes (A, blue color).

Supplementary Figure 3:

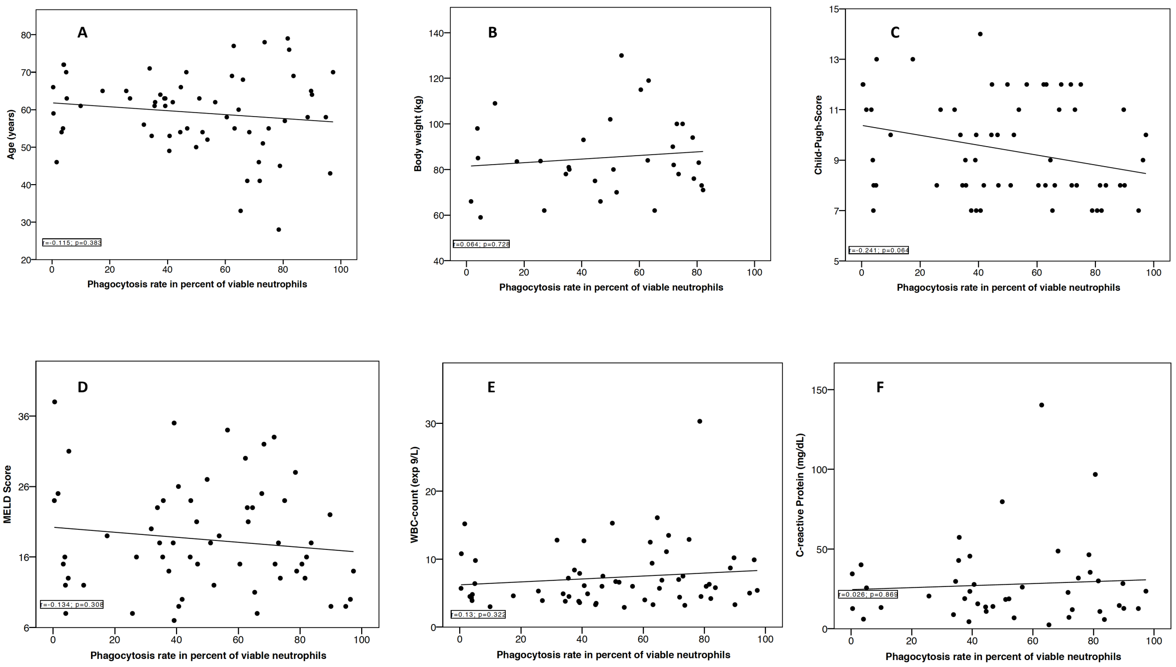

Correlation between phagocytic rate in ascites neutrophils and systemic factors: A) age, B) body weight, C) Child-Pugh-Score, D) MELD, E) WBC count, F) CrP.

Supplementary Figure 4:

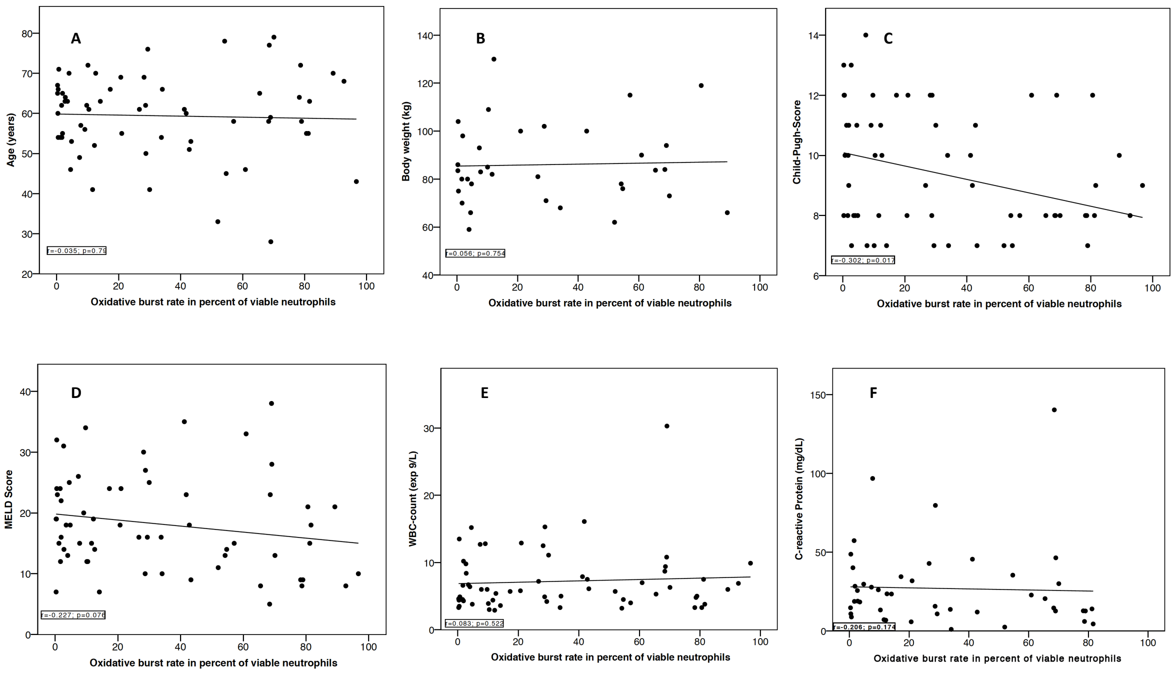

Correlation between oxidative burst rate in ascites neutrophils and systemic factors: A) age, B) body weight, C) Child-Pugh-Score, D) MELD, E) WBC count, F) CrP.

Tables

Supplementary Table 1: Influence of drugs on the phagocytic rate and oxidative burst rate of ascites neutrophils

Drug	Phagocytic rate (%), median (range)	Level of significance (p)	Oxidative burst rate (%), median (range)	Level of significance (p)
Proton pump inhibitors (administration vs. no administration)	46.5 (0.4-97.3) vs. 52.1 (0.5-94.8)	0.824	17.3 (0.3-96.7) vs. 28.7 (0.5-81.6)	0.33
Antibiotic prophylaxis (administration vs. no administration)	38.2 (0.4-78.9) vs. 55.15 (0.5-97.3)	0.117	11.1 (0.3-98.2) vs. 28.45 (0.3-96.7)	0.614
Beta blocker (administration vs. no administration)	51 (0.4-90) vs. 49.9 (0.5-97.3)	0.948	11 (0.3-92.7) vs. 31.6 (0.4-96.7)	0.065
Diuretics (administration vs. no administration)	43.1 (0.4-96.3) vs. 58.5 (0.5-97.3	0.233	15.7 (0.3-96.7) vs. 28.5 (0.5-89.2)	0.291
Lactulose (administration vs. no administration)	44.5 (0.4-78.5) vs. 55.15 (0.5-97.3)	0.14	14.1 (0.3-89.2) vs. 29.4 (0.3-96.7)	0.249
Human albumin (administration vs. no administration)	46.5 (0.4-96.3) vs. 53.8 (0.5-97.3)	0.574	23.9 (0.3-96.7) vs. 35 (0.4-81.2)	0.773

Supplementary Table 2: Correlation between phagocytic rate and oxidative burst rate of blood neutrophils and systemic factors.

Factor	Phagocytic rate (r)	Level of significance (p)	Oxidative burst rate (r)	Level of significance (p)
Age (years)	0.032	0.808	-0.028	0.829
Body weight (kg)	0.217	0.217	-0.32	0.061
Child-Pugh score	-0.057	0.659	-0.102	0.428
MELD score	-0.225	0.079	-0.278	0.028
WBC (exp9/L)	-0.185	0.149	-0.27	0.032
CrP (mg/dL)	-0.057	0.714	-0.34	0.022
Serum protein (mg/L)	0.076	0.699	0.036	0.856
Serum albumin (g/L)	0.094	0.468	-0.149	0.253

WBC – White blood cell count

CrP - C-reactive protein
